# Nursing Home’s Measures during the COVID-19 Pandemic: A Critical Reflection

**DOI:** 10.3390/ijerph19010075

**Published:** 2021-12-22

**Authors:** Hongli Sam Goh, Vivian Tan, Chen-Na Lee, Hui Zhang, M Kamala Devi

**Affiliations:** 1 Nursing Administration, Kwong Wai Shiu Hospital, Singapore 328127, Singapore; 2 Lee Ah Mooi Nursing Home, Singapore 168871, Singapore; vuivui.tan@gmail.com; 3 Department of Internal Medicine, Singapore General Hospital, Singapore 168753, Singapore; chenna_lee@hotmail.com; 4 Alice Lee Centre for Nursing Studies, Yong Loo Lin School of Medicine, National University of Singapore, Singapore 117597, Singapore; nurzh@nus.edu.sg (H.Z.); nurmkd@nus.edu.sg (M.K.D.)

**Keywords:** long-term care, community nursing, COVID-19, coronavirus, workforce, SARS-CoV-2, pandemic

## Abstract

This study examined the pandemic measures taken by nursing leaders to cope with COVID-19 at a nursing home in Singapore. The pandemic has affected over 215 countries, sparking a series of containment and pandemic measures by governments and healthcare organizations worldwide. Long-term care facilities are especially vulnerable to the pandemic, but little has been reported about the nursing homes’ measures in handling the pandemic. The present study used Morley’s (2014) three-stage critical reflection method to review meeting minutes, organizational emails, and government advisories on the COVID-19 pandemic measures undertaken by nursing leaders at a nursing home in Singapore between January and June 2020. The pandemic measures were broadly classified into four groups: (1) infection surveillance and containment measures; (2) ensuring continuity in clinical care and operational support; (3) resource and administrative coordination; and (4) staff training and development. Nurses have played a vital role in the fight against COVID-19 by ensuring continuity in patient care and demonstrating clinical leadership in pandemic efforts. This study proposes a useful nursing pandemic structure that outlines a set of functions and measures required for handling a pandemic and that can be applied to various medical emergencies and contingencies.

## 1. Introduction

In December 2019, a novel coronavirus, COVID-19, emerged and has resulted in a pandemic affecting over 215 countries worldwide within a short span of five months. As of 30 June 2021, 181,521,067 people have been infected globally, with 3,937,437 deaths [1]. Singapore reported its first COVID-19 case on 23 January 2020. Due to rapid local community transmission, the country raised its Disease Outbreak Response System Condition (DORSCON) alert level to orange on 7 February 2020. On 7 April 2020, the country introduced “circuit breaker” measures to stamp the sharp rise in COVID-19 cases within the community [2]. Due to the unknown pathology of the disease, its high transmission rate, and asymptomatic infections, this pandemic sparked a flurry of clinical guidelines put forth by governments and healthcare organizations worldwide to contain its spread and impact [3].

Long-term care facilities are especially vulnerable to infectious diseases due to their residents’ profile, infrastructure constraints, manpower and resource shortages, and limited government funding [4]. These systemic challenges have contributed to reports of COVID-19 transmission within nursing homes in the United States, the United Kingdom, and Singapore [5,6,7]. In Singapore, the majority of the nursing homes resemble dormitory-style residential conditions with shared communal facilities and close proximity among residents [8]. The physical layout, coupled with the systemic challenges nursing homes face, could provide the impetus for the COVID-19 outbreak. Tan and Seetharaman reported high rates of acute respiratory symptoms among nursing home residents, making it difficult for clinicians to differentiate COVID-19 cases from non-COVID-19 ones [9]. 

Management guidelines for the COVID-19 outbreak can be more complex for nursing homes than those of acute hospitals due to differences in physical layout, resources, and residents’ disease profiles [9]. In a local news report, a nursing home operator had to resort to seeking assistance and additional manpower from the Ministry of Health (MOH) to support its daily operations when several of its staff and residents were infected with COVID-19 and required to quarantine. Staff shortages and lean resources have been identified as contributory causes [10]. Although governmental bodies have been swift in introducing pandemic guidelines, success is contingent on the extent and pace of implementation by nursing homes. While there have been several published reports on COVID-19 pandemic measures taken by acute hospitals and primary and tertiary care centers [11,12,13], little has been reported about measures taken in the long-term care sector. Therefore, there is a need to shed light on the efforts and measures taken by nursing leaders to manage COVID-19 within nursing home settings.

## 2. Materials and Methods

This study examined the pandemic measures taken by nursing leaders to cope with COVID-19 at a charitable home in the long-term care sector in Singapore. The study employed Morley’s three-stage critical reflection method involving data immersion, deconstruction, and reconstruction to generate recommendations for practice [14,15]. The method is based on several theoretical traditions, including Schon’s reflective practice, critical postmodernism theory, and discourse analysis. The researchers chose this method as it was helpful in exploring assumptions and actions within the social context and re-interpreting their meaning for renewed options for societal action [15]. 

The study was conducted at a 624-bedded charitable nursing home in central Singapore. In Singapore, there are a total of 16,221 beds across 77 nursing homes, which fall into three categories: public (*n* = 24, 31.1%), private (*n* = 31, 40.3%), and charitable/not-for-profit (*n* = 22, 28.6%) [16]. Most public and charitable nursing homes receive substantial government subsidies, operate under donor funding, and require co-payment from clients. On the contrary, as private nursing homes are not under the MOH subsidy scheme, clients are required to make direct out-of-pocket payments [17]. The majority of the public and charitable nursing homes’ layout resembles dormitory-style living conditions shared between 6 and 12 residents, who are mostly elderly and require a moderate to high level of care assistance [17]. However, private nursing homes vary widely in living conditions, from single- or double-bedded private rooms to a dormitory with as many as 30 residents [17]. 

According to Morley’s (2014) three-stage critical reflection approach, the first stage involved two researchers (G.H.S. and V.T.) collating documents, making field notes about the pandemic measures at the nursing home, and immersing themselves in the data from January to June 2020 [14]. The second stage involved deconstruction, with the two researchers discussing and generating evolving concepts [14]. They analyzed various data sources, such as meeting minutes, organizational emails, and government advisories. Finally, the third stage involved reconstruction, with the researchers grouping the final concepts into categories with critical incidents as supporting examples and proposing a set of actions for change [14]. The study’s trustworthiness was ensured by prolonged data immersion and member checking of notes [15]. 

The study was reviewed by the institutional management and exempted from ethics approval, as it did not involve any human subjects. Permission was given by the management team to report and publish the findings of the study.

## 3. Results

The pandemic measures adopted by the nurse leaders at the nursing home are classified into four groups: (1) infection surveillance and containment measures; (2) ensuring continuity in clinical care and operational support; (3) resource and administrative coordination; and (4) staff training and development. These measures, which have continually evolved in response to the MOH’s national directives and the DORSCON alert level, are discussed in the following section.

### 3.1. Infection Surveillance and Containment Measures

In January 2020, when news of the COVID-19 outbreak was reported in China, the nursing home set up a command center and a nursing taskforce committee to monitor the situation. The Nursing Director and the Infection Control Nurse (ICN) chaired the nursing taskforce committee and sat in the command center to oversee the nursing home’s pandemic response and coordination efforts. They managed the setup of the screening counter and the surveillance system for staff/visitor traffic movement and served as the subject matter expert for the command center. The ICN was also responsible for reviewing national pandemic guidelines; consulting other experts; and liaising with the MOH, other government agencies, and healthcare organizations. “*The ICN would provide a daily update and submission of data to MOH* via *AIC [Agency for Integrated Care]. She would work with the Clinical Director to collate the information from other non-nursing departments. They will disseminate any information to the rest of the nursing home*” (MM1, Infection Control Nurse).

The ICN’s role proved pivotal to the nursing home’s capability to cope with the pandemic measures. She mobilized the necessary resources and support from the nursing workforce at short notice. For example, the ICN had to review and contextualize government guidelines into organizational directives for each department. “*We [ICN and Nursing Director] need to translate the MOH DORSCON alert level into organization-specific directives on infection prevention measures for frontline and admin staff*” (JN1, Infection Control Nurse).

The ICN also worked with the nurse clinician to coordinate mask-fitting exercises for over 400 staff within three weeks to ensure that they were prepared for the COVID-19 pandemic measures. As a result, when the COVID-19 cases spiked in May 2020 with widespread community transmission in Singapore, the nursing home had already instituted organizational guidelines on pandemic measures and established vital infrastructures to cope with the pandemic per the MOH directives.

### 3.2. Ensuring Continuity in Clinical Care and Operational Support

When Singapore implemented the DORSCON Orange alert level in February 2020 to contain small clusters of COVID-19 transmission within the community, the nursing home intensified manpower and resource planning efforts to ensure continuity in clinical practice care and operational support. To ensure sufficient manpower, the nurse managers informed all frontline staff to defer non-essential overseas trips. In addition, they explored alternative staffing arrangements, such as spilt-team/spilt-site arrangement, a 12 h shift rotation, and an extension of work hours. “*The nursing home will be implementing the “split-team; split-site” arrangement for all non-essential staff*” (OE1, Nursing Director). A ward-specific staffing threshold was also set for the possible activation of additional staffing in the event of high work absenteeism. To sustain adequate resources for clinical operations, nurse managers projected ward utilization rates for essential resources, such as personal protective equipment (PPE). Areas for the consolidation of services were also identified, resulting in the delegation of non-essential tasks to non-clinical staff and the suspension of certain services. The Nursing Director mentioned, “Non-clinical staff will be deployed and assigned to man certain areas such as registration counter or visitor escort” (OE1).

The nurse managers played a crucial role in ensuring continuity of care for the residents. They had to increase ward round frequencies and provide constant communication with staff and oversee their compliance with the prevailing infection control directives, which were continuously changing at a rapid pace. They also assessed vulnerable residents with acute respiratory symptoms for medical referral, screened residents’ outpatient appointments, and rescheduled non-essential ones to minimize residents’ movement out of the nursing home. Nurse Manager 4 said: “*We are doing more frequent ward rounds to comb the wards to make sure that there are no sick residents with ARI [acute respiratory infection]*” (JN2). The constant presence of the nurse managers at the frontline also helped to ensure rapid information dissemination, staff compliance to pandemic directives, and the monitoring of staff safety and welfare during this trying period. 

The nursing team also relied on information and communication technology to substitute face-to-face family visits with remote visitation and important meetings with web-based conferencing. During a meeting, the Nursing Director reported: “*The Admission team has used Zoom to facilitate tele-visitation between our residents and family members*” (MM5). These measures assisted the nursing home in maintaining communication with residents’ next of kin and friends during the pandemic.

### 3.3. Resources and Administrative Coordination

Two non-nursing administrative staff members assisted the nursing taskforce committee in logistics and administrative and manpower support functions, which centered on inventory management, organizational and documentation support, and data collation required for government reporting. The administrative staff assisted the Nursing Director in conducting business continuity planning for the entire nursing home, such as spilt-site and spilt-team work arrangements and identifying areas for service consolidation. They also assisted the ICN in data collation for mandatory reporting to the MOH. As data were primarily in hardcopy format, collating such information can be challenging and time-wasting. The Infection Control Nurse commented: “*J and M [pseudonyms] continue to support me ICN in gathering information based on the MOH’s demand as collating such information from different departments at short notice at the behest of the MOH can be technically draining for me*” (MM2). The administrative staff proved valuable in helping the clinicians with mundane tasks, thus freeing them to focus on coordination and communication efforts with various governmental agencies. 

Other than operational and administrative support, the administrative staff also supported the nurse managers in overseeing human resource matters, such as staff welfare and lodging. Nurse Manager 2 said during a meeting: “*We must be physically present to make sure our staff are okay, and they know we are there for them. If they are stressed, we can always activate H.R. to support them, whether it is finance or emotional issue.*” (MM2). When workplace segregation was instituted to minimize staff movement within the 624-bedded facility, the administrative staff coordinated with the human resource personnel to ensure similar living arrangements within the nursing home dormitory for the foreign staff to minimize risks for cross-cluster transmission. Temporary accommodations were also arranged if foreign staff were issued quarantine orders or evicted from rental housing by their landlords. Other resources, such as a helpline for psychological support and food catering, were also arranged to ensure staff welfare and mental well-being. Nursing Director: “*Management and H.R. should pay more attention to staff welfare. The following measures may be considered: Provide recognition for staff in small tangible ways, e.g., words of encouragement, surprise pack, subscribe to entertainment channels on T.V. for stay-in staff to watch in-house*” (GA1).

### 3.4. Staff Training and Development

The last function pertains to the specific training needs of nursing staff to handle the pandemic. This function was assigned to the nurse educator to coordinate and conduct essential training, such as upskilling the long-term care nurses’ competencies in pandemic management. At the behest of the Agency for Integrated Care, the nurse educator was tasked with helping to organize mask-fitting train-the-trainer courses for the long-term care sector, including the general practitioners, clinic staff, and other nursing homes that might not have the capabilities to support such efforts. 

When Singapore introduced the “circuit breaker” measures, a tighter set of safe distancing measures, in April 2020 to stem the spike in COVID-19 cases, many training providers were restricted from conducting face-to-face training, forcing many essential training programs, such as cardiopulmonary resuscitation and the use of personal protective equipment, to cease temporarily. These measures prompted the training department to revamp their courseware for online delivery, such as recording procedural skills and teleconferencing platforms to enable continuous learning. Nurse Educator: “*The education team has no choice but to stop [classroom] training and convert them into online learning. This will allow us to continue to train our staff*” (OE5). At the same time, the nurse educator also worked with the training team to rapidly build its training infrastructure, such as the learning management system. The nurse educator commented on how the COVID-19 pandemic had a positive aspect in regard to expediting the nursing home’s use of technology to continue operations and training. As a result, the nurse educator converted her training materials to electronic format and utilized web-based communication technologies to deliver her staff training to more staff. 

## 4. Discussion

The creation of the nursing taskforce committee, which consists of nurse leaders and the ICN, proved valuable in the early establishment of communication channels and the coordination of infection surveillance and containment efforts at the nursing home. As the government continues to roll out new directives during the COVID-19 situation, the structure and functions of the nursing taskforce committee have evolved, resulting in its existing framework. In this framework, the nursing taskforce committee plays a crucial role in reviewing the latest pandemic guidelines and advising the command center on clinical operations and resource management as subject matter experts. In addition, the Nursing Director coordinates internal communication to keep all stakeholders updated on government advisories on the COVID-19 situation. At the same time, the ICN serves as the primary contact point for external communication with the MOH, other government agencies, and healthcare organizations. 

**Figure 1:**
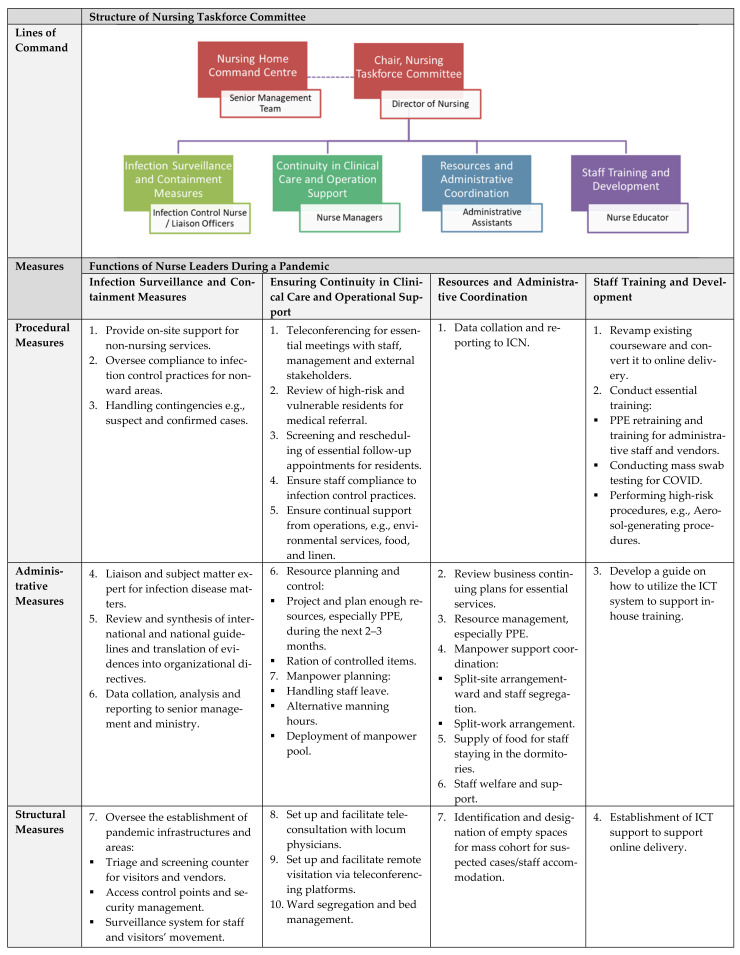

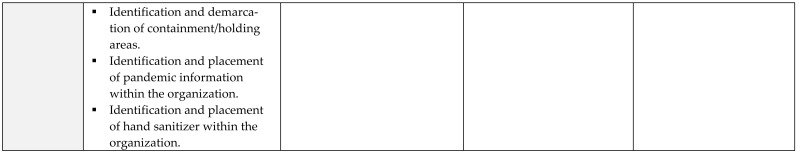
Pandemic nursing framework at the nursing home.

The current nursing pandemic framework has provided the nurse leaders with a highly efficient approach to handle the COVID-19 situation at the nursing home (Figure 1). The Nursing Director and ICN assume the infection surveillance and containment measures function and direct the nursing taskforce committee in collaboration with the command center to set the overall direction and efforts for the pandemic. The ICN oversees the surveillance and containment measures, such as staff/visitor’s movement, infrastructures, and security points. During the pandemic, two key issues emerged for the nursing taskforce committee: (1) the exigency of data collation for the real-time monitoring of suspected/confirmed cases and (2) the review of multiple COVID-19 reports of pandemic responses. In our experience, the establishment of a dynamic pandemic response system is lacking in the long-term care sector due to heterogeneity in organizational systems and processes, limited funding and governmental support in infrastructure development. Paterson et al. highlighted the daunting task of reviewing the overwhelming number of COVID-19 reports published almost daily, much less developing effective pandemic strategies accordingly [18]. This laborious but necessary process has resulted in the nursing home dedicating two nurse leaders to assist the ICN in the data collation and review of the latest COVID-19 directives from the government [18].

The nurse managers assumed the function of ensuring continuity in clinical care and operational support, such as manpower and resource planning, clinical care coordination, documentation, and environmental control. The two non-nursing administrative staff members assisted the nursing taskforce committee with resources and administrative coordination by focusing on inventory management, organizational support, and data collation. These concerted efforts allowed the nursing taskforce committee to implement government-issued directives in a timely and effective manner and the nursing home to ensure sufficient resources for daily operations despite a supply crunch. For example, there were reports of healthcare organizations facing shortages in PPE, disinfectants, and other essential items worldwide, prompting the World Health Organization to issue advisories on the prudent use of PPEs during the COVID-19 pandemic [19,20]. 

The nurse educator assumed the staff training and development function by conducting essential training and upskilling the long-term care nurses’ competencies in pandemic management. She also contributed to the development of the online training infrastructures to ensure continuity in essential staff training. Several studies have highlighted the importance of staff upskilling and cross-training to enable their competencies to handle the pandemic and deployment to critical work areas when needed [9,21,22]. At the time of this writing, the nursing home has generated sufficient training infrastructure and resources to offer their in-house long-term care training courses to other nursing homes using online delivery. 

This study reports the current framework to provide nurse leaders with pandemic management strategies. Although some have considered pandemics a natural disaster and considered the use of disaster nursing models to inform disaster risk reduction strategies, the pandemic presents a different set of challenges, involving a less hazardous but more dynamic situation than other natural disasters [21]. There are currently limited studies to guide nursing functions and measures during a pandemic. In several studies on pandemic measures within nursing homes, Stall et al. detailed the different phases of pandemic measures outlining the various responses implemented to stabilize an outbreak situation in Toronto, Canada. At the same time, Yen et al. described the use of environmental scanning and control to mitigate COVID-19 risks in Taiwan [23,24]. In the U.S. and Europe, several studies outlined the challenges long-term care facilities faced and proposed a series of measures to be taken to protect these organizations during the COVID-19 pandemic [25,26]. Our study contributed to the literature by providing nursing home leaders with a better understanding of the structures, functions, and measures required for handling future pandemic situations, particularly in data collation, communication, and resource management.

## 5. Conclusions

Being the largest workforce in healthcare, nurses have played a vital frontline role in the fight against COVID-19. They have demonstrated clinical leadership by maintaining care continuity, directing pandemic efforts, and building organizational capabilities to handle the crisis. This study highlighted several challenges nurse leaders faced in the long-term care sector during a pandemic, requiring a multi-prong, collaborative, and coordinated approach involving various stakeholders. As the long-term care setting presents unique challenges, international and national guidelines need to be contextualized into the sector-specific and organizational directives before they can be implemented successfully. 

The present study proposes a useful nursing pandemic structure that outlines a set of functions and measures required for handling a pandemic and that can be applied to various medical emergencies and contingencies. The structure can also be used to guide the curriculum and develop core competencies in pandemic management for nurses in the long-term care sector. Nurse educators can also utilize the framework to develop training materials to improve the staff’s capability in handling pandemics in the future.

## Data Availability

The data presented in this study are available on request from the corresponding author. The data are not publicly available due to privacy restrictions.
